# The Online Elicitation of Personal Utility Functions (OPUF) tool: a new method for valuing health states

**DOI:** 10.12688/wellcomeopenres.17518.1

**Published:** 2022-01-14

**Authors:** Paul P. Schneider, Ben van Hout, Marike Heisen, John Brazier, Nancy Devlin

**Affiliations:** 1 School of Health and Related Research, University of Sheffield, Sheffield, UK; 2 OPEN Health, York, UK; 3 Open Health, Rotterdam, The Netherlands; 4 School of Population and Global Health, University of Melbourne, Melbourne, Australia

**Keywords:** EQ-5D, Health valuation, multi-attribute value theory, multi-criteria decision analysis, online survey, personal utility function, preference elicitation, stated preferences

## Abstract

Introduction

Standard valuation methods, such as TTO and DCE are inefficient. They require data from hundreds if not thousands of participants to generate value sets. Here, we present the Online elicitation of Personal Utility Functions (OPUF) tool; a new type of online survey for valuing EQ-5D-5L health states using more efficient, compositional elicitation methods, which even allow estimating value sets on the individual level. The aims of this study are to report on the development of the tool, and to test the feasibility of using it to obtain individual-level value sets for the EQ-5D-5L.

Methods

We applied an iterative design approach to adapt the PUF method, previously developed by Devlin et al., for use as a standalone online tool. Five rounds of qualitative interviews, and one quantitative pre-pilot were conducted to get feedback on the different tasks. After each round, the tool was refined and re-evaluated. The final version was piloted in a sample of 50 participants from the UK. A demo of the EQ-5D-5L OPUF survey is available at: https://eq5d5l.me

Results

On average, it took participants about seven minutes to complete the OPUF Tool. Based on the responses, we were able to construct a personal EQ-5D-5L value set for each of the 50 participants. These value sets predicted a participants' choices in a discrete choice experiment with an accuracy of 80%. Overall, the results revealed that health state preferences vary considerably on the individual-level. Nevertheless, we were able to estimate a group-level value set for all 50 participants with reasonable precision.

Discussion

We successfully piloted the OPUF Tool and showed that it can be used to derive a group-level as well as personal value sets for the EQ-5D-5L. Although the development of the online tool is still in an early stage, there are multiple potential avenues for further research.

## 1 Introduction

The valuation of health, in terms of quality-adjusted life years (QALYs), is an essential component in health economic evaluations. The QALY is generally derived from generic measures of health, which, in turn, consist of two components: firstly, a health descriptive system, which defines a number of mutually exclusive health states and, secondly, a set of (social) values, that reflect their respective desirability. These values are commonly based on individual preferences of members of the general public ^1, 2^.

Methods for eliciting preferences belong to one of two types: they are either compositional or decompositional ^3– 5^. Standard health state valuation methods, such as time trade-off (TTO), standard gamble (SG), discrete choice experiments (DCE) and best-worst scaling (BWS) belong to the latter group. Their main disadvantage is that they are inefficient. The amount of information that is obtained from each participant is so small, that data from hundreds, if not thousands, of participants is required in order to estimate a social value set. Generating value sets for small subgroups will thus often not be feasible at all ^6, 7^.

Compositional methods, on the other hand, are much more efficient – they even allow the estimation of value sets on the individual-level. Values can also directly be aggregated across individuals, without the need for complicated statistical models. Nevertheless, compositional methods have seldom been used in the valuation of health and, where they have been used, it is generally in combination with decompositional methods ^8^.

Recently, Devlin *et al.* ^9^ pioneered a new method for eliciting health state values, based entirely on compositional preference elicitation techniques. Their personal utility function (PUF) approach was successfully piloted in face-to-face interviews to derive personal (as well as a social) value sets for the EQ-5D-3L instrument ^10^. The EQ-5D-3L is a generic measure of self-reported health, which is widely used in health economic evaluations (see below).

In this paper, we aim to expand on the previous PUF work in three ways. Firstly, we establish its theoretical foundations, namely multi-attribute value theory, and how it relates to the valuation of health states more generally (section 2). Secondly, we report on the development of a new, PUF-based online tool (OPUF) to obtain individual-level value sets for the EQ-5D-5L (section 3), and then pilot the tool in a small sample of participants (section 4). Finally, we discuss the main advantages, disadvantages, and potential challenges, and propose potential next steps in the development of the OPUF approach (section 5).

## 2 Theoretical framework

Preference-based measures of health are (implicitly or explicitly) built on multi-attribute value or utility theory (MAVT/MAUT). These frameworks provide the theoretical foundations for the application of compositional and decompositional preference elicitation methods ^11– 13^. Before we provide a brief introduction into MAVT/MAUT, it may useful, however, to highlight some relevant aspects of health descriptive systems, to demonstrate how closely they are linked to MAVT/MAUT.

### 2.1 Health descriptive systems

Most health descriptive systems, generic or condition-specific, share a similar structure, in the sense that health states are defined along a set of dimensions (e.g. pain, mobility, etc), of which each has a number of attributes, reflecting different levels of performance ^1, 14^. These levels usually have an inherent order, such that higher levels are preferred over lower level, or vice versa (e.g. some pain is better than severe pain). All possible combinations of attributes from different dimensions define the complete set of health states that a descriptive system can represent. Moreover, in most systems there is one best state, full health, which dominates all other states, and one worst state, which is dominated by all other states. For use in health economic evaluations, health descriptive systems need to be valued: utility values, anchored at full health (=1) and dead (=0), need to be assigned to all health states. These values are sometimes also referred to as social values, preference-based indices and health utilities , (health-related) quality of life-, or QALY-weights (we use these terms synonymously). As we will explain below, the structure of a health descriptive system is crucial for its valuation.

### 2.2 The EQ-5D-5L instrument

To give an example, and also to describe the instrument that is to be valued in this study using the OPUF, we briefly introduce the EQ-5D-5L ^15^. This health descriptive system defines health states using five dimensions/criteria: mobility (MO), self-care (SC), usual activities (UA), pain or discomfort (PD), and anxiety or depression (AD). Each dimension has five performance levels: no, slight, moderate, severe, and extreme problems. However, the extreme level for dimensions MO, SC; and UA use the word ‘unable’ (e.g. unable to walk about). In total, the instrument describes 3,125 mutually exclusive health states. They can be referred to by a 5-digit code, representing the severity levels for the five dimensions. ‘11111’ denotes full health; and ‘55555’ denotes the objectively worst health state.

### 2.3 Multi-attribute value and utility theory

MAVT and MAUT are general (multi-criteria decision making) frameworks to analyse decision problems involving multiple alternatives and conflicting objectives. The difference between MAVT and MAUT is that the former deals with problems under certainty, while the latter also incorporates uncertainty. The general concept, however, is the same: the stated preferences of an individual, or a group of individuals, over a number of alternatives can be quantified as a value (or utility) function, which assigns a score to any alternative under consideration. The alternatives only have value in so far as they meet certain objectives. This makes it possible to learn a decision maker’s partial preferences for these objectives, construct a preference function, and then use it to predict values for different alternatives ^3, 5^.

The valuation of health states can be described with this framework ^13^. The three general structural levels (alternatives, objectives, performances) can be mapped directly to corresponding concepts in health descriptive systems. Firstly, the alternatives under consideration, which are to be valued, correspond to health states. Secondly, the objectives against which alternatives are to be evaluated correspond to the different health dimensions (e.g. pain, mobility). Thirdly, the alternatives’ performance levels, i.e. the extent to which the alternatives meet the objectives, correspond to the dimension levels of the different health states (e.g. some pain, impaired mobility, etc).

### 2.4 Value measurement theory

In the context of the QALY framework, constructing a value function for health states requires three components:

1. **Level ratings/scores**: also referred to as marginal value functions, reflect the preferences for different levels of performance on a given criterion. This specifies, for example, how much better *some* pain is compared to *severe* pain. The scale is defined by the best and worst possible level of performance. The units of measurement are arbitrary, but for convenience, values are usually normalised between 100 (best) and 0 (worst).
2. **Criteria/dimension weights**: they represent the relative importance of a given criterion, compared to all other criteria. More specifically, it is a measure of the relative (utility) gain associated with replacing the lowest level with the highest level of performance for this criterion (e.g. moving from extreme pain to no pain). A value of 100 is assigned to the most important criterion, and the weights of all other criteria are then defined relative to this yardstick: a value of 50, for example, means a criterion is half as important; a value of zero means a criterion is not important at all.
3. **Anchoring factor**: anchoring is an additional step, only required in the context of the QALY framework. It is necessary, because health state utilities need to be mapped on to a scale, which is anchored at full health, set to 1, and dead, set to 0. For this, an additional parameter needs to be elicited, that we will call anchoring factor ^16^. It was operationalised as a person’s maximum range of utility values, i.e. the difference between their highest and their lowest utility value. Alternatively, it can be understood as a person’s (assumed) rate of substitution between units of quantity and units of quality of life.

All three components are combined into a (global) value function, using some pre-specified aggregation method. Most commonly, an additive aggregation function (weighted sum) is chosen. It is easy to interpret, as it only considers marginal changes. Since we want to anchor utility values on the QALY scale, we first need to normalise the additive function between 1 and 0 (i.e. divide both components by 100), and then rescale the function, using the anchoring factor *a*. Accordingly, an additive model with *m* criteria can be written as:


$$V(h)=1-a*\sum_{i=1}^{m}1-\frac{w_{i}p(h_{i})}{1000}$$

whereby *V*( *h*) is the value function which assigns a utility value to any health state *h*; *a* is the anchoring factor (=utility range); *w _i_* is the weight of the *i*th dimension, *h _i_* is the level of performance of state *h* on criterion *i*, and *p*( *h _i_*) then gives the marginal value of state *h*’s performance level on dimension *i*. It should be noted that the anchoring factor is usually not explicitly considered as a separate criterion in the value function. Instead, it is used to rescale the dimension weights and level ratings (see section ’How to construct PUF’s from participants’ responses’ below).

### 2.5 Decompositional and Compositional methods

As stated in the introduction, there are two types of preference elicitation methods: compositional and decompositional methods. We assume that readers will be familiar with decompositional methods, in the form of TTO, SG, DCE, or BWS. All of these methods require participants to evaluate entire health states. This means, they need to consider all the relevant criteria at the same time, and then assign cardinal values to these states. Subsequently, these values are decomposed, with the aim to work out the marginal contribution of each attribute to the overall utility score. Ultimately, this procedure provides a scoring system, with coefficients for the different dimensions and levels, which can be used to estimate the values for all health states.

Another aspect that should be noted is that, in practice, it is usually infeasible to elicit values for all health states from one individual. Therefore, a statistical model needs to be fitted to the values elicited from multiple individuals over a subset of the states ^17, 18^. Depending on the complexity of the health descriptive system, large numbers of participants may need to be surveyed to yield sufficient data points for the statistical model to converge and to produce robust estimations ^6, 7^. This makes it generally impossible to construct value functions for small groups or for single individuals.

The elicitation of preferences through compositional methods works the other way around. They start with the valuation of the individual components of health states: criteria weights, level ratings and the anchoring factor are elicited directly and in separate tasks. The three components are then combined, using a pre-specified aggregation function, to estimate the values for all health states.

There are several compositional preference elicitation techniques that can be used ^4^. The most straight-forward methods involve asking participants to allocate points or rate the attributes directly, using a visual analogue scale (VAS), for example. Alternative methods include ranking techniques, Likert-type scales (AHP) or semantic categories (MACBETH) ^19– 21^.

These techniques have been used extensively in multicriteria decision analysis (MCDA), including numerous applications in the context of health technology assessments ^22– 24^. Up until now, however, the application of compositional methods in health valuation studies has been scarce. One notable exception is the Health Utility Index (HUI 2, HUI 3) ^8, 25^. Based on a MAUT framework, value sets were derived by combining the (decompositional) SG method with a (compositional) visual analogue scale. Criteria weights and the anchoring factor were (simultaneously) derived through the former, while the latter provided the levels scores. However, the PUF approach appears to be the first that is entirely based on compositional preference elicitation techniques ^9^.

## 3 Development of the OPUF Tool

### 3.1 From PUF to OPUF

The PUF approach was developed by Devlin *et al.* ^9^ as a new method to derive personal value sets for the EQ-5D-3L ^10^. It consists of a series tasks, organised in seven sections (A: warm-up, B: dimension ranking, C: dimension rating, D: level rating, E: paired comparison, F: position-of-dead, G: check for interactions). The approach was successfully piloted in 76 face-to-face interviews. The results showed that compositional methods can be used to derive EQ-5D-3L value set on the group, as well as on the individual level.

In recent years, the use of online data collection of stated preferences data has become more and more popular. The main reasons for this are presumably the speed and the often markedly reduced costs compared to interviewer administration. This may, in part, also explain the rise in the use of DCE, which, compared to TTO, are much easier to apply online ^26, 27^.

The aim of the present study was to adapt and refine the PUF approach for use as a stand-alone online survey, and to test its use in valuing the EQ-5D-5L. With one exception (G: check for interactions) all tasks used in the original approach were implemented in the OPUF. We only added one additional task, the ’Dead-VAS’, to be able to anchor the PUF of participants with a certain preference profile (see below). Nevertheless, the overall implementation of the OPUF differed significantly from the original. The original PUF approach was delivered in face-to-face interviews. Participants were encouraged to reflect on, explain, and revise their responses. Deliberation and the interaction with the interviewer were key components of the study, and interviews took up to 90 minutes. We believe this approach cannot easily be replicated in a stand-alone online tool. Participants may be less motivated to work through difficult exercises or to reflect on their preferences, without the presence of a human interviewer. We therefore decided to make the survey shorter, and focused on clear and intuitive presentation of the tasks. For this, we simplified some of the instructions and tried to design an easy-to-use web interface.

### 3.2 Development of the EQ-5D-5L OPUF Tool

The OPUF Tool was programmed in R Shiny – an extension of the R programming language for creating interactive user interface ^28^. For the development, we used an iterative design approach. First, we experimented with various approaches for emulating the PUF tasks, that were applied in face-to-face interviews conducted online survey. This involved exploring the capabilities of R Shiny, and testing different input elements, such as numeric or text input fields, buttons, drop-down menus, and sliders. Since default templates did not always seem adequate, we developed several new input elements, including visual analogue scales (VAS), a level rating scale, and a colour-coded DCE. Different presentations of the tasks were discussed among the research team and tested with colleagues. Three different versions of the online tool were built before we developed a first fully functional prototype.

Subsequently, the prototype was evaluated and further refined in five iterative rounds of user testing. This involved qualitative online interviews with a total of 22 participants (5+4+4+5+4), recruited via the Prolific platform ( https://www.prolific.co). During the interviews, we observed the participants’ screens while they were going through the OPUF Tool. After each task, we asked them how they understood the task, how difficult it was, and whether there was anything confusing about it. The interviews took between 15 and 53 minutes. After each round, we revised the tool based on the feedback we received. After the third round, we also conducted a first ’test launch’, for which we recruited 50 participants to complete the tool without being directly monitored by the interviewer. Data from the test launch was used to check and refine our analysis plan.

Once we arrived at the final version of the OPUF Tool, we conducted a quantitative pilot to test the feasibility of using it for deriving personal as well as group-level EQ-5D-5L utility functions. The results are described in section 4 (quantitative pilot results).

### 3.3 The EQ-5D-5L OPUF Tool

The OPUF Tool consists of 10 steps. In the following, we describe each step in more detail and explain how the respective tasks work. However, we consider the visual presentation of the tasks an essential component of the OPUF Tool. Much effort went into developing an intuitive and easy-to-use design. We thus recommend readers to consult the online demo version of the tool while reading through this section. It is available at https://eq5d5l.me.

***Steps 1 & 2: Warm-up***

The first two tasks aim to familiarise participants with the instrument and the five dimensions it covers. They are asked to self-report their current health on the EQ-5D-5L descriptive system and to rate their overall health status, using the EQ-VAS. To avoid any anchoring effect, we designed a new, empty slider input element, which had no default value.

***Step 3: Level rating***

In the original PUF, level rating involved five separate tasks, one for each dimension of the EQ-5D-3L. Participants were asked to allocate 100 points between an improvement from extreme to moderate, and from moderate to no problems. Since no and extreme problems are fixed at 100 and 0, in effect, this exercise determined the values of the ‘moderate’ level on each dimension. For the OPUF Tool, the move from the 3L to the 5L version meant that we had to reconsider the design. Asking participants, for each dimension, to allocate points to four improvements (extreme to severe, severe to moderate, moderate to slight, and slight to no problems) seemed excessive. We thus considered two alternative options:

A Use the design for the 3L version to elicit a score for the moderate level on each dimension, and then linearly interpolate the scores for the slight and severe level. This assumes that the differences between levels are equal.
B Elicit scores for all levels without any reference to a particular dimension. This assumes that the different levels of severity (‘slight’, ‘moderate’ etc.) have consistent interpretations, irrespective of the specific health problem.

We assessed the model coefficients of existing EQ-5D-5L value sets from different countries, to check whether either of the options could be supported by empirical data. However, the evidence was ambiguous and partly contradictory. Ultimately, we chose to implement option B (elicit all level ratings without reference to a specific dimension) because it seemed more convenient for the participants.

The final instructions for the task state that “a person with 100% health has no”, and “a person with 0% health has extreme health problems”. Participants are then asked: "[h]ow much health does a person with slight health problems have left?". Responses are recorded on a scale that ranges from 100% (= no problem) to 0% (extreme problems). After the participant clicks on the scale, two things happen. Firstly, the label (’slight problems’) and a connecting arrow appear right next to the selected value; and secondly, the question changes to the next severity level (i.e. from slight to moderate, and from moderate to severe). The severity levels are highlighted, using a purple background colour (the hue depends on the severity level).

During the entire pilot phase, this task was considered to be difficult by many of the participants. Especially in earlier versions of the tool, participants were often confused by the instructions and we had to revise and simplify the instructions and layout several times.

In a previous version, the task also included default values, i.e. the values of slight, moderate, and severe problems were preset to 75%, 50% and 25%, respectively, and participants were asked to adjust them. Yet, this caused a strong anchoring effect and many participants did not change those values: 26 of 50 participants (52%) kept the preset value for the moderate severity level, for example. Adapting the design, so that it did not show any defaults, was technically challenging, but seemed necessary in light of these early findings.

***Step 4: Dimension ranking***

Participants are presented with the worst levels of each dimension (i.e. ‘I am unable to walk about, I am unable to wash and dress myself, etc), and asked to rank them in order of which problem they would ‘least want to have’; ties were not permitted. The task aims to introduce participants to the idea of prioritising one dimension of health over another. Responses to this task are also used to tailor the presentation of the following task to the individual participant.

***Step 5: Dimension weighting (Swing weighting)***

Five sliders are shown, one for each dimension, describing an improvement from the worst (extreme problems) to the best level (no problems). The sliders are presented in the same order as the participant had just ranked them. The first slider, for the most important dimension, is set to 100. This is given as a fixed yardstick, that participants are asked to use to evaluate the relative importance of the improvements in the other dimensions (which are set to 0 by default).

The instructions are tailored to each participant: if, for example, extreme pain or discomfort was ranked first in the previous task, the instructions state: “If an improvement from ‘I have extreme pain or discomfort’ to ‘I have no pain or discomfort’ is worth 100 ’health points’, how many points would you give to improvements in other areas?”.

***Step 6: Validation DCE***

Three pairwise comparisons between health states are sequentially presented to the participant: they are asked whether they prefer scenario A or B. The health states for the scenarios are personalised. For each participant, the dimension weights and the level ratings are combined into a (1-0 scaled) PUF. This function is then used to value all 3,125 health states, and to establish a preference order. Ties are broken randomly.

Health states for scenario A are selected from the 25th, 50th, and 75th percentile (order randomised) of the participant’s personal ranking. The scenario A states are then paired with states that have an absolute utility distance of about 0.1 (hard choice), 0.2 (medium choice), and 0.3 (easy choice), respectively (order randomised). Dominated and dominating states are excluded.

To make it easier for participants to asses the severity of a health state, we used intensity colour coding, i.e. different shades of purple were used as background colours, ranging from light purple for no problems to dark purple for extreme problems, as previously suggested by Jonker *et al.* ^29^.

The responses to this task were not used in the construction of the PUF – the purpose was to assess how accurately the OPUF approach can predict an individual participant’s actual choices in a standard discrete choice experiment task.

***Step 7: Position-of-Dead Task***

In this task, participants go through up to six paired comparisons between A) a health state and B) ’Being Dead’. In the first comparison, scenario A is the worst health state (‘55555’). If the participant prefers that state over dead, the participant immediately proceeds to Step 8. If they prefer dead, a binary search algorithm is initiated, to find the state that is equal to dead.

As before, in Step 6, the participant’s individual PUF is used to value and rank all 3,125 health states. After the participant’s indicated that state ’55555’ is worse than being dead, the search goes to the median state. From there, it moves up or down, depending on the participant’s choices, in half-intervals. The search stops after five iterations. At this point, the equal-to-dead state is identified with a maximum error of +/- 49 states, corresponding to 1.6% of the total number of states defined by the EQ-5D-5L.

In a previous version of the tool, the dead state was labelled ‘Immediate Death’. Through the qualitative interviews, however, we learned that this made many participants think about the process of dying and they were consequently rather hesitant to ever choose this option. We changed the label to ‘Being Dead’. We also decided not to display any duration for scenario A, because in the QALY framework, utility independence must be assumed.

***Step 8: Dead-VAS***

Those participants, who indicated they would prefer the worst health state (’55555’) over being dead, are asked to assess the value of that health state on a vertical visual analogue scale. The top anchor point, at 100, is labelled ’No health problems’, and the bottom, at 0, is labelled ’Being Dead’. The description of the worst health state is shown in a box next to the scale. When the participant selects a position value, an arrow is displayed, connecting the box to the respective position on the scale.

A previous version of the tool did not include the Dead-VAS, but instead all participants completed three TTO tasks: two warm-up tasks and then one TTO involving the worst health state. However, this design often lead to inconsistent responses: 19 of 50 participants (38%) reversed their preference between the Position-of-Dead and the TTO task. More specifically, 15 (30%) switched from worst health state ≺ dead to dead ≻ worst health state, while 4 (8%) switched the other way around. Although smaller, the latter group was more problematic, because their responses made it impossible to anchor their PUFs, at all.

The inconsistent results could be attributable to several factors. First of all, it is a well known (and unavoidable) fact that different valuation techniques yield different utility values, and thus different anchor points [ 1, p. 49–76]. Other potential explanations might include differences in the interpretation of the tasks, the additional consideration of time (displayed in the TTO, but not in the Position-of-Dead task), or lack of attention.

To ensure that PUFs can be constructed for all participants, we decided to implement the Dead-VAS. The task also appeared to be easier for the participants and also quicker to complete (the TTO took more than 2 minutes, i.e. 20% of the average completion time, in the pre-pilot).

***Step 9: demographics***

This step includes questions about personal characteristics that are assumed or have shown to explain some of the variability in people’s health preferences, including age, partnership status, sex, having children, nationality, importance of religion, spirituality or faith, and the frequency of engaging in religious activities, level of education, work status, income, and experience with poor health ^10, 30^.

### Add-on: Personal results page

As a thank-you, some of the PUF results are fed back to the participants at the end of the survey. Presented are the dimension ranking and the level rating tasks, as well as estimated utility values for four different health states. Participants could compare their results with aggregate results from the overall sample of participants in each study, and with the value sets for EQ-5D-5L obtained from the English general population using conventional decompositional methods, as reported by Devlin *et al.* ^9^.

Most participants found it difficult to interpret the results; the meaning of the health state values were unclear. Notwithstanding, many participants appreciated the results page, if only as a gesture, and found it interesting to compare their own results with those from the general population.

### Other learnings from the qualitative pilot

The online interviews played a key role in the development of the OPUF Tool. The feedback from participants helped us to identify many minor and major issues, and the tool underwent significant changes over the course of the pilot. The changes affected almost every aspect, including the wording of questions, the presentation of the tasks, the overall layout, and the mechanics of different tasks.

A main challenge in the development process was to strike the right balance between rigour/completeness and ease of use. For example, we started with long descriptions for all tasks, which often included examples, and some also contained animations (e.g. to demonstrate how sliders work). We realised, however, that when descriptions were too long or complicated, participants would skip over them and/or disengage with the tasks. We therefore gradually shortened the descriptions and simplified the language. Overall this seemed to be more effective in conveying the relevant information. The final version only contains very short instructions, and we sought to apply an intuitive design, which eliminates the need for elaborate explanations.

Through the pilot we also learned that from interactions with other websites, most people have developed very clear expectations about interacting with online surveys. When elements (such as buttons, sliders, etc) were presented in a slightly unusual way, it often caused confusion and participants sometimes got stuck on a task. To give just one example, in a previous version, the OPUF Tool included a text box next to a visual analogue scale. The text box would show the value that the participant selected on the scale. At the beginning (when the participant had yet not selected a value), however, the box would be empty. This led several participants to assume that they were expected to enter a value into the box manually. They tried to click on it and to type in a number. Since this did not work, they got frustrated and it took them a while until they realised they had to use the scale instead. This problem was easily resolved by just hiding the box in the beginning, and only showing it after the participant had clicked on the scale and selected a value. In another context, we implemented loading animations, to draw the participants’ attention to specific parts of the page when they changed. Otherwise, participants often did not notice that a new task had already started and they were waiting for something to happen. These small ’tricks’ very much helped to improve the user experience, which seemed suboptimal, in earlier versions of the OPUF Tool.

The usability of the final version received very positive feedback, and participants described it as "easy to navigate", "clear", or "easy to red and understand". One participant stated that "it felt like everything clicked into place".

## 4 Quantitative pilot results

We conducted a quantitative pilot study to assess the feasibility of OPUF Tool in practice. As for the qualitative pilot, recruitment was conducted through the Prolific platform without any restrictive inclusion criteria or quota – any adult person from the UK with a prolific account could participate. The main points of interest were the plausibility of the responses, the consistency across tasks, and the participants’ engagement with the online tool. We also tested our methods of analysis: the collected preference data was used to construct individual and social value functions, and to value all 3,125 EQ-5D-5L health states. We did not attempt any further exploratory or confirmatory analysis of the data, since this was only a pilot study, without a representative sample.

### Sample

Fifty participants were recruited. Of these, 23 (46%) were younger than 30 years of age, 18 (36%) were between 30 and 39, and 9 (18%) were 40 years of age or older. Thirty (60%) participants were female, 20 (40%) were male. A majority of 32 (64%) participants had a high level of education (degree or post-graduate).

***Step 1+2: Warm-up***

Fourteen (28%) participants reported to be in perfect health. The remaining 36 (72%) participants also mostly reported slight or moderate health problems. Self-reported health on the visual analogue scale ranged from 100 to 40, with a mean (SD) and median (IQR) of 78 (14) and 80 (21.25), respectively.

***Step 3: Level ratings***

**Figure 1.**
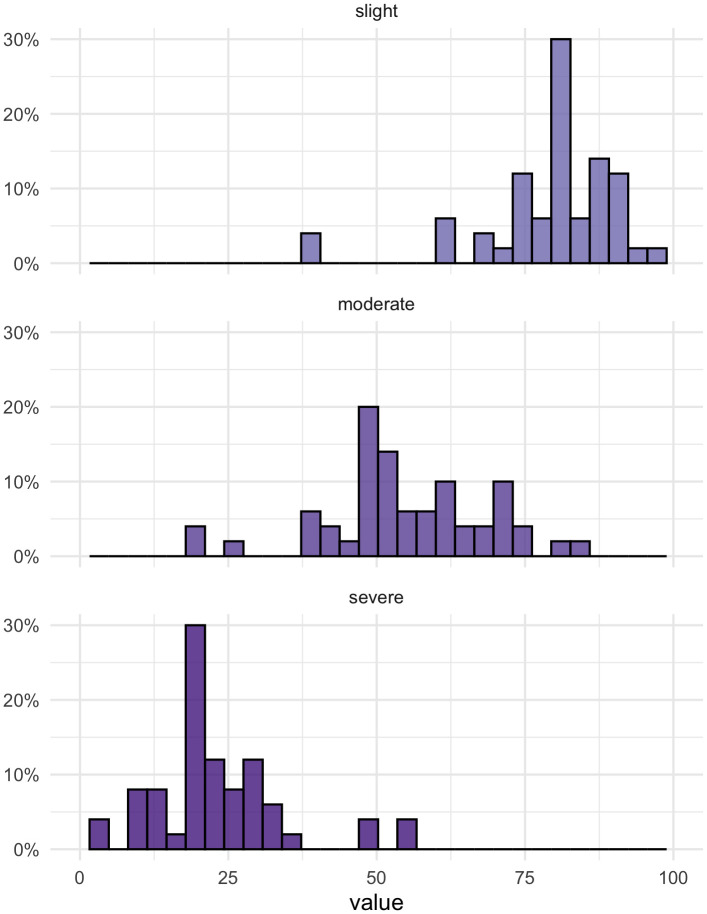
Level ratings for ’slight’, ’moderate’, and ’severe problems’.

Mean (SD) ratings for the level slight, moderate, and severe were 79.10 (11.45), 54.92 (13.41), and 23.46 (11.27) (the ratings of no and extreme problems were fixed at 100 and 0). Figure 1 shows the full distributions of values assigned to the three levels.

Forty (80%) and 41 (82%) participants set their own values for the slight and severe levels, i.e. they changed the default values. For the moderate level, only 26 (52%) changed the value, which may be an indication for the presence of an anchoring effect.

***Step 4: Dimension ranking***

Table 1 shows the results of the ranking exercise. Twenty-three (46%) participants considered Pain/Discomfort the most most important criterion. The average ranking of this dimension was 2.2. It was followed by Mobility (mean rank = 2.4), Self-Care (3.0), Anxiety/Depression (3.6), and, lastly, Usual Activities (3.8).

**Table 1.** Summary of the dimension ranking exercise.

Rank	MO	SC	UA	PD	AD
1 ^*st*^	15 (30%)	8 (16%)	1 (2%)	23 (46%)	3 (6%)
2 ^*nd*^	14 (28%)	11 (22%)	7 (14%)	8 (16%)	10 (20%)
3 ^*rd*^	10 (20%)	14 (28%)	12 (24%)	7 (14%)	7 (14%)
4 ^*th*^	9 (18%)	9 (18%)	10 (20%)	10 (20%)	12 (24%)
5 ^*th*^	2 (4%)	8 (16%)	20 (40%)	2 (4%)	18 (36%)

MO = Mobility; SC = Self-Care; UA = Usual Activities; PD = Pain/Discomfort; AD = Anxiety/Depression

***Step 5: Dimension weighting (swing weighting)***

**Figure 2.**
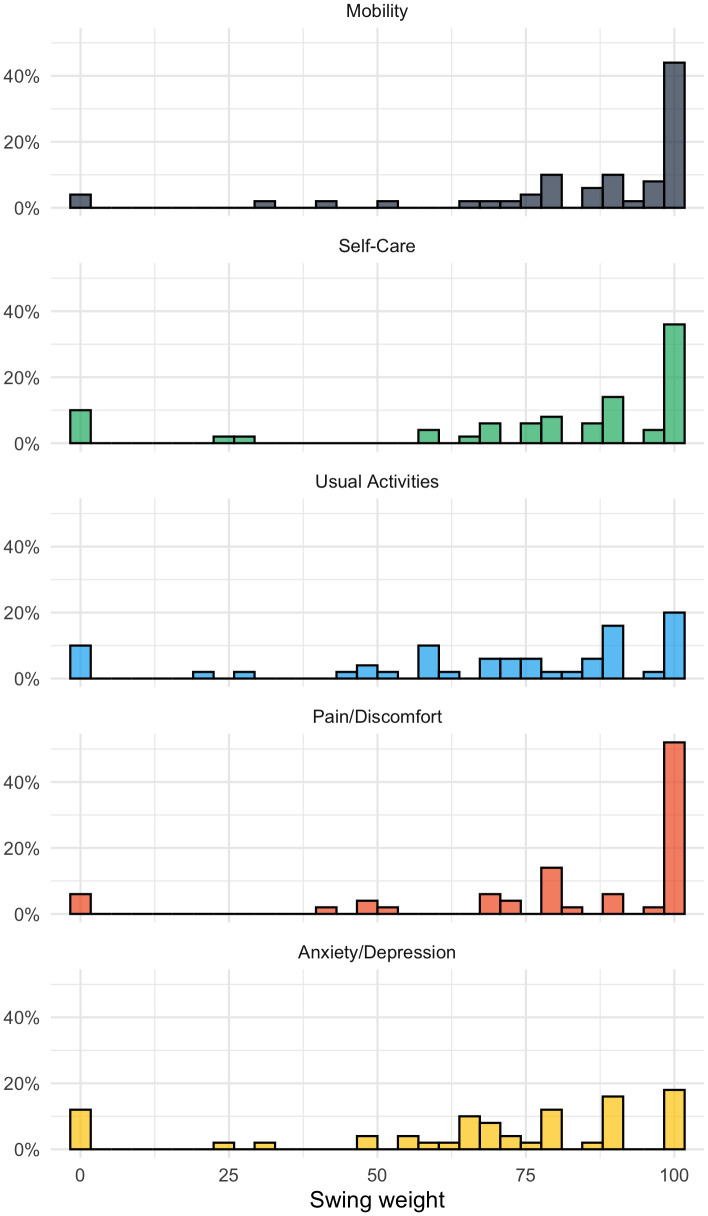
Swing weights for dimension MO = Mobility, SC = Self-care, UA = Usual activities, PD = Pain/discomfort, AD = Anxiety/depression.

Figure 2 shows the distribution of the weights assigned to the five EQ-5D-5L dimensions. The dimension with the highest mean (SD) weight was Mobility at 85.16 (23.51), followed by Pain/Discomfort at 83.08 (26.41), Self-Care at 77.38 (30.22), Usual activities at 69.78 (30.22), and then Anxiety/Depression at 67.78 (30.78). Four (8%) participants assigned a value of 100 to all dimensions; 7 (14%) assigned a value of zero to one or more dimensions.

The weights of 30 (60%) participants implied different preference order, i.e. at least one preference reversal, compared to the order specified in the previous ranking task (ties were not considered an order violation). As noted above, these inconsistencies do not necessarily signify that participants did not pay attention. In the qualitative pilot, some participants deliberately chose a different ranking, in response to the slightly differently phrased question.

***Step 6: Validation DCE***

Each participant completed three paired comparisons. Of the 150 choices, 120 (80%) were consistent with the choices predicted by participants’ PUFs. More specifically, 28 (56%) participants made no inconsistent choice, 15 (30%) made one, six (12%) participants made two, and one (2%) participants made three ’errors’.

We also found that the larger the utility difference between the two states in a choice set, the smaller the error rate: at a distance of about 0.1 (on a normalised 0-1 scale, dominating/dominated states were excluded), the error rate was 26%, at 0.2, it was 24%, and at 0.3, it was 10%.

***Step 7: Position-of-Dead Task***

A total of 18 (36%) participants stated that they would prefer the worst health state state (‘55555’) over ’being dead’. Another nine (18%) preferred ’being dead’ in the first choice set, but then choose the health state in the next five sets. Of the remaining participants, the position of dead varied greatly. The number of states considered worse than dead ranged from 0 (0%) to 2,883 (92%), with a mean and median of 483 (15%) and 50 (2%).

***Step 8: Dead-VAS***

The 18 participants, who considered the worst health state better than ’being dead’, completed the Dead-VAS task. Their valuations of the worst health state on a scale between 100 (’no health problems’) and 0 (’being dead’) ranged from 5 to 70, with a mean (SD) and median (IQR) of 23.22 (21.03) and 19.5 (21.75).

***Step 9: Demographics***

Some of the collected demographic information (age, sex, level of education) are provided above in the description of the study sample. Further data are not reported here, since this is only a pilot study, and we did not attempt to make any inferences about participants personal characteristics.

### Survey duration

On average, it took participants about seven minutes (range: 3.6 – 18.2 mins) to complete all tasks. The longest time (76 secs) participants spent completing the survey was on the dimension weighting task and the demographic questions. The shortest duration was observed for the subjective health status (EQ-VAS) (21 seconds). Further details on the time participants spent on different tasks are shown in Table 2. With only very few exceptions (e.g. one participants spent only 4 seconds on the dimension ranking task), the observed times seemed by and large plausible and suggested that participants did engage with the tasks.

**Table 2.** Survey completion times (in seconds).

	Mean	SD	Min	25th perc.	Median	75th perc.	Max
Own Health State	29	17	11	18	23	30	96
EQ-VAS	21	18	6	11	15	24	116
Level Rating	58	33	17	36	49	66	177
Dimension Ranking	51	33	4	33	41	58	184
Dimension Weighting	76	47	18	50	62	89	274
Validation DCE	63	27	20	45	57	70	165
Position-of-Dead Task	48	34	7	17	44	64	172
Dead-VAS (conditional)	26	12	15	17	22	32	56
Demographics	76	26	43	62	72	85	195
Total	431	178	215	318	356	508	1091
Total (Minutes)	7.2	3.0	3.6	5.3	5.9	8.5	18.2

### How to construct PUFs from participants’ responses?

Constructing a participant’s PUF required two steps: firstly, level ratings were combined with the dimension weights. Secondly, the resulting model coefficients were anchored on to the QALY scale.

In the first step, level ratings, ranging from 100 (no problems) to 0 (extreme problems) were converted to disutilities, ranging from 0 (no problems) to 1 (extreme problems). For convenience, dimension weights were also normalised so that the sum of all five weights summed up to 1. By taking the outer product of these two vectors, we derived a (1-0 scaled) set model coefficients.

In the second step, these coefficients were anchored on the QALY scale, using either the state that was determined to be approximately equal to ’being dead’ in the position-of-dead task (for 32 participants who considered one or more health states worse than ’being dead’), or the value that was assigned to the worst health state (’55555’) in the Dead-VAS task (for the other 18 participants).

To illustrate the computation with a simple example: suppose an individual rated the five severity levels (denoted *l*) in the following way: *l _no_* = 100, *l _slight_* = 90, *l _moderate_* = 50, *l _severe_* = 30, and *l _extreme_* = 0. Furthermore, they assigned the following weights (denoted *w*) to the five dimensions: *w _MO_* = 100, *w _SC_* = 60, *w _UA_* = 45, *w _PD_* = 80, and *w _AD_* = 70. After converting to level ratings to disutilties and normalising the weights, we get the following two vectors:


$$l=\left[\begin{array}{c} \;0 \end{array}\;\begin{array}{c} 0.1 \end{array}\;\begin{array}{c} 0.5 \end{array}\;\begin{array}{c} 0.7 \end{array}\;\begin{array}{c} 1 \end{array}\;\right];\;w=\left[\begin{array}{c} \;0.29 \end{array}\;\begin{array}{c} 0.1 \end{array}7\;\begin{array}{c} 0.11 \end{array}\;\begin{array}{c} 0.23 \end{array}\;\begin{array}{c} 0.2\; \end{array}\right]$$

Taking the outer product provides a (scaled) matrix $\tilde{M}$ , containing all 25 level-dimension coefficients (see below). These coefficients can already be used to value (on a 0-1 scale) and rank health states. The value for ’12345’, for example, is 1 − (0 + 0.02 + 0.06 + 0.16 + 0.20) = 0.56. It should be noted that this procedure is also used within the OPUF Tool, in order to determine the algorithm for the Position-of-Dead and also to select choice sets for the DCE validation task.


$$\begin{array}{l} \;\begin{array}{ccccc} w_{MO} & \;w_{SC} & \;w_{UA} & \;w_{PD} & \;w_{AD} \end{array}\\ l⊗w=\tilde{M}=\begin{array}{r} \begin{array}{r} l_{no}\\ l_{slight}\\ l_{moderate}\\ l_{severe}\\ l_{extreme} \end{array} \end{array}\left(\begin{array}{c} 0\\ 0.03\\ 0.14\\ 0.20\\ 0.29 \end{array}\;\begin{array}{c} 0\\ 0.02\\ 0.09\\ 0.12\\ 0.17 \end{array}\;\begin{array}{c} 0\\ 0.01\\ 0.06\\ 0.08\\ 0.11 \end{array}\;\begin{array}{c} 0\\ 0.02\\ 0.11\\ 0.16\\ 0.23 \end{array}\;\begin{array}{c} 0\\ 0.02\\ 0.10\\ 0.14\\ 0.20 \end{array}\right) \end{array}$$

Suppose that for this individual, the health state ’51255’ was identified as being approximately similar to being dead in the Position-of-Dead task. After we compute the (scaled) disutility for state ’51255’ (= 0.29 + 0 + 0.02 + 0.23 + 0.2 = 0.74), we can anchor and rescale the coefficient matrix, by simply dividing it by this value:


$$\begin{array}{l} \;\begin{array}{ccccc} \;w_{MO} & \;w_{SC} & \;w_{UA} & \;w_{PD} & \;w_{AD} \end{array}\\ \frac{\tilde{M}}{0.74}=M=\begin{array}{r} \begin{array}{r} l_{no}\\ l_{slight}\\ l_{moderate}\\ l_{severe}\\ l_{extreme} \end{array} \end{array}\left(\begin{array}{c} 0\\ 0.04\\ 0.19\\ 0.27\\ 0.39 \end{array}\;\begin{array}{c} 0\\ 0.02\\ 0.12\\ 0.16\\ 0.23 \end{array}\;\begin{array}{c} 0\\ 0.02\\ 0.08\\ 0.11\\ 0.15 \end{array}\;\begin{array}{c} 0\\ 0.03\\ 0.15\\ 0.22\\ 0.31 \end{array}\;\begin{array}{c} 0\\ 0.03\\ 0.14\\ 0.19\\ 0.27 \end{array}\right) \end{array}$$

Now, we have derived the individual’s PUF. It sets ’51255’ to 0 (1 − (0.39 + 0 + 0.02 + 0.31 + 0.27) = 0); ’11111’ is still equal to 1 (1−(0+0+0+0+0) = 1), and the worst health state (’55555’) is set to -0.35 (1 − (0.39 + 0.23 + 0.15 + 0.31 + 0.27) = −0.35).

### Individual and social PUF

We constructed PUFs for all 50 participants. The descriptive statistics are provided in Table 3. The first column shows the mean coefficients. These mean values may also be taken as the group-level value set (i.e. the group tariff). The 95% confidence intervals were bootstrapped using 10,000 iterations. The width of the confidence intervals suggests that, even with a small sample size of only 50 participants, the OPUF approach allowed us to estimate a group tariff with reasonable precision.

**Table 3.** Descriptive statistics for 50 PUFs (i.e. personal model coefficients).

Dim	Lvl	Mean (95% CI)	Min.	25 ^*th*^ perc.	Median	75 ^*th*^ perc.	Max.
MO	2	0.072 (0.064; 0.099)	0.000	0.031	0.048	0.083	0.573
	3	0.150 (0.138; 0.188)	0.000	0.075	0.126	0.185	0.679
	4	0.250 (0.234; 0.302)	0.000	0.137	0.219	0.309	0.793
	5	0.344 (0.316; 0.437)	0.000	0.175	0.282	0.354	1.554
SC	2	0.057 (0.053; 0.070)	0.000	0.027	0.045	0.076	0.207
	3	0.121 (0.112; 0.151)	0.000	0.068	0.099	0.160	0.622
	4	0.207 (0.192; 0.258)	0.000	0.139	0.176	0.242	1.057
	5	0.282 (0.254; 0.375)	0.000	0.167	0.247	0.309	2.073
UA	2	0.051 (0.047; 0.063)	0.000	0.020	0.040	0.069	0.166
	3	0.103 (0.097; 0.124)	0.000	0.055	0.090	0.144	0.357
	4	0.182 (0.170; 0.221)	0.000	0.102	0.174	0.213	0.629
	5	0.234 (0.219; 0.281)	0.000	0.131	0.219	0.265	0.761
PD	2	0.062 (0.057; 0.078)	0.000	0.030	0.051	0.079	0.281
	3	0.132 (0.123; 0.160)	0.000	0.067	0.114	0.159	0.500
	4	0.225 (0.211; 0.273)	0.000	0.138	0.185	0.269	0.840
	5	0.291 (0.274; 0.351)	0.000	0.173	0.249	0.339	1.000
AD	2	0.052 (0.046; 0.071)	0.000	0.020	0.042	0.066	0.413
	3	0.104 (0.096; 0.130)	0.000	0.045	0.093	0.133	0.489
	4	0.175 (0.163; 0.213)	0.000	0.092	0.154	0.201	0.572
	5	0.231 (0.214; 0.288)	0.000	0.124	0.205	0.259	1.086

MO = Mobility; SC = Self-Care; UA = Usual Activities; PD = Pain/Discomfort; AD = Anxiety/Depression

**Figure 3.**
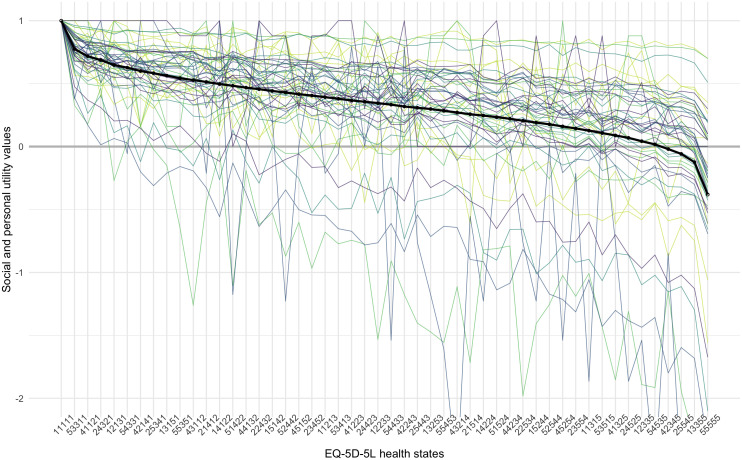
Personal and group-level utility functions for 50 health states, ordered from best to worst, according to the group preference. The thick lines represent the group preference, and the thin lines represent the 50 underlying personal utility functions. The different colours are used to distinguish between separate individuals and have no other meaning.

Figure 3 illustrates all 50 personal, as well as the average, group-level utility function for a small subset set of EQ-5D-5L health states. Shown are the values for 50 health states, ranked 1 ^*st*^, 65 ^*th*^, 129 ^*th*^, 192 ^*th*^, 256 ^*th*^, ..., 3125 ^*th*^, according to the group-level utility function.

It can be seen from the graphs that health state preferences of the participants differed considerably. Two separate processes can be distinguished: firstly, lines depicting personal utility values go up and down, and cross each other, while the group preference is monotonically decreasing. This illustrates individual differences in the relative ranking of health states. Secondly, the range of utility values also varies greatly between participants. For some participants, all health states have high values, within a narrow range, while for others, the range of utility values is much wider. Accordingly, the value of the worst health state (’55555’) ranges from a maximum of 0.7 to a minimum of -3.2, with a mean and median of -0.4 and -0.2. For comparison, the population estimate reported by Devlin *et al.* is -0.285 ^18^.

It may be interesting to note the difference between the mean and the median, as it shows the effect that outliers, with a wide utility range, have on the overall group tariff. This is not an uncommon finding in valuation studies and for the construction of a social value set, one may want to consider following the common practice of rescaling the negative values to have a lower limit of -1, or using the median, instead of the mean, to aggregate preferences across individuals ^31^.

## 5 Discussion

This study provides a comprehensive description of the new OPUF Tool. It covers the theoretical background, reports on the iterative development, and provides a pilot study, which demonstrates that it is feasible to use the online tool for eliciting personal, as well as group-level, preferences for EQ-5D-5L health states.

We think the OPUF Tool provides a flexible, conceptually attractive, and potentially useful new approach for deriving value sets for the EQ-5D-5L (or any other health descriptive system). It could be used as a standalone solution, or to complement established (decompositional) methods, by providing more detailed preference information. The compositional preference elicitation techniques included in the OPUF Tool have several advantages over the more commonly used decompositional methods, which may make the approach particularly attractive to other researchers.

In contrast to the TTO, which is generally administered in face-to-face interviews (though can be online), the OPUF is applied online, which makes it easier and cheaper to collect preference data. The qualitative feedback received during the online interviews even suggests that participants tended to find the online survey to be interesting and engaging. Furthermore, the OPUF approach provides value sets which are anchored on the QALY scale (i.e. at full health and dead), and not only on a latent scale (i.e. un-anchored), which is usually the case in conventional DCE surveys.

Another advantage of the OPUF approach over other conventional valuation methods is the statistical power: fewer participants are required to derive a group tariff or social value set. Note that even with data from just 50 participants, we were able to derive relatively precise estimates for an EQ-5D-5L group tariff. The OPUF Tool may thus allow estimating value sets for smaller groups (e.g. local communities, patient groups), which could practically not be estimated using decompositional methods.

As we have demonstrated, utility functions can even be estimated on the individual-level. This enables researchers to investigate the heterogeneity of health state preferences between individuals in an unprecedented level of detail. It could potentially be useful for other applications beyond health economics (e.g. individualised cost-effectiveness analyses ^32^). For example, the OPUF approach could be used as a patient decision aid and to facilitate shared decision making in a clinical context. Explicitly weighing different aspects of health might help patients, who face complex treatment decisions, to better understand the trade-offs that are involved, and what aspects are most important to them.

Furthermore, we would like to draw attention to the fact that the calculations required to construct individual and group-level preferences in the OPUF approach are relatively simple. This makes the underlying model more transparent and potentially easier to communicate to decision makers than more sophisticated statistical models, such as a mixed conditional logit, or a Bayesian hybrid model ^18, 33^.

Finally, another benefit of compositional preference elicitation techniques may be that they break down the valuation of health states into sub-tasks (level rating, dimension weighting, anchoring). The original PUF approach made use of this and encouraged participants to reflect on their preferences at every step of the survey. The OPUF Tool could also be adapted for this purpose and be applied in computer-assisted personal interviews. A study that uses a modified version of the tool to facilitate deliberative discussions among groups of participants is currently under way.

This study also has several important limitations that need to be considered.

Firstly, in the development of the OPUF Tool, ’ease of use’ was a main goal. Some valuation tasks were thus simplified, in order to reduce the burden for the participants. For example, we used a single level rating task for all dimensions combined, instead of having separate tasks for each. This assumes the that the relative positions of slight, moderate, and severe problems are the same across all five EQ-5D dimensions. In the absence of any authoritative guidance, it remains unclear whether we struck the right balance between rigour and ease of use.

Secondly, every task has a design which shapes how participants respond to it and which may influence their decision making. This is referred to as choice architecture ^34^. Further evaluation of the OPUF Tool could help to assess to what extent participants’ responses are sensitive to changes in the presentation of the different tasks, and to improve the quality and robustness of the survey.

Thirdly, an important limitation of compositional preference elicitation techniques is that they cannot easily be used to test for interaction effects. Rather, a functional form must be assumed a priori. In our study, we assumed an additive, main effects model. This seemed reasonable, because it is commonly used to represent health state preferences – most EQ-5D-5L value sets are based on such a model. When studies test for and include interaction effects, authors also often find only minor improvements in the explanatory power ^35^.

Finally, some important challenges of the OPUF Tool are likely not methodological, but normative. Over the last decades, decompositional preference elicitation methods, have been used extensively in the valuation of health and are by now well established. The compositional methods, used in the OPUF Tool, on the other hand, are new. Decision makers may be less familiar with them, and they may also appear to be conceptually different. This raises the question, *are the derived value sets equally valid*?

Assessing validity of a new method for valuing health is an intricate problem, as there is no gold standard against which it could be compared. At the moment, several valuation methods (SG, TTO, DCE, etc) are used side by side, and numerous studies have shown that these different methods, and even variations of the same method, produce different results ^1, 36– 38^. It is not clear, which, if any, of these methods should be considered to be *the best*. Nevertheless, the findings from this study indicate at least a high level of consistency between the OPUF approach and DCE. We included three standard DCE tasks in the survey and found that the constructed PUF of a particular participant predicted their choices in a DCE task with an accuracy of 80%.

Irrespective of the comparably high level of agreement with DCE, some readers may argue that eliciting preferences requires observing choices involving trade-offs and potentially also risk and uncertainty. Compositional techniques may then seem principally inappropriate. To this, we would reply that MAVT/MAUT provide broad theoretical frameworks, on the basis of which different methods can be justified. Moreover, deviations from formal (Welfare) economic theory are common in health economics and other areas. Simplifications are often made to make certain applications practically feasible. The QALY framework, for example, can be viewed as a major simplification, yet it proved to be immensely useful to inform resource allocation in health care. Similarly, the OPUF Tool may be based on a simpler conception of individual preferences, but it enables new types of analyses (e.g. preferences heterogeneity) and makes it possible to derive value sets on the individual level and in settings in which it would otherwise be unfeasible (e.g. small patient groups).

### Next steps

The immediate next step will be to replicate the pilot in a larger study, not only to show that the OPUF can be used to estimate a country-specific social tariff, but also to demonstrate how information on individuals’ personal preferences can be harnessed to investigate the heterogeneity of preferences between individuals and/or societal subgroups.

Furthermore, it should be noted that the OPUF approach is not specific to the EQ-5D instrument. The approach is, in principle, applicable to any health descriptive system. This might be true not only on the conceptual level, but also on the technical: the OPUF Tool was programmed in R/Shiny ^28^. For the implementation, we developed several generic methods and input elements. This means, the tool could quickly be adapted for different settings (e.g. other country) or instruments (e.g. SF-6D) ^39^. Several steps in the development could then be automated. With some further abstraction, the underlying code could potentially provide a flexible, modular software platform for creating valuation tools for any health descriptive system.

## Conclusion

Using an iterative design approach, we developed the OPUF Tool; a new type of online survey to derive value sets for the EQ-5D-5L. Based on compositional preference elicitation techniques, it allows the estimation not only of social, but also of personal utility functions. In this study, we successfully tested the OPUF Tool and demonstrated its feasibility in a in a sample of 50 participants from the UK. Even though the development is still in an early stage and further refinement is required, we see several potential applications for the OPUF approach.

## Data availability

### Underlying data

Zenodo: bitowaqr/opuf *_d_emo*: *OPUF zenodo version 1*, *https://doi.org/10.5281/zenodo.5773915*.

Data are available under the terms of the Creative Commons Zero "No rights reserved" data waiver (CC0 1.0 Public domain dedication).

## Ethical approval

The study was approved by the Research Ethics Committee of the School of Health and Related Research at the University of Sheffield (ID: 030724). We obtained written informed consent from all participants for the use and publication of their data.

## References

[1] Brazier J Ratcliffe J Saloman J : Measuring and valuing health benefits for economic evaluation.OXFORD university press,2017. 10.1093/med/9780198725923.001.0001

[2] Whitehead SJ Ali S : Health outcomes in economic evaluation: the QALY and utilities. *Br Med Bull.* 2010;96:5–21. 10.1093/bmb/ldq033 21037243

[3] Keeney RL Raiffa H Rajala DW : Decisions with Multiple Objectives: Preferences and Value Trade-Offs. *IEEE Transactions on Systems, Man, and Cybernetics.* 1979;9(7):403. 10.1109/TSMC.1979.4310245

[4] Marsh K IJzerman M Thokala P : Multiple criteria decision analysis for health care decision making--emerging good practices: report 2 of the ISPOR MCDA Emerging Good Practices Task Force. *Value Health.* 2016;19(2):125–137. 10.1016/j.jval.2015.12.016 27021745

[5] Belton V Stewart T : Multiple criteria decision analysis: an integrated approach.Springer Science & Business Media,2002. 10.1007/978-1-4615-1495-4

[6] Gandhi M Xu Y Luo N : Sample size determination for EQ-5D-5L value set studies. *Qual Life Res.* 2017;26(12):3365–3376. 10.1007/s11136-017-1685-3 28825183

[7] de Bekker-Grob EW Donkers B Jonker MF : Sample size requirements for discrete-choice experiments in healthcare: a practical guide. *Patient.* 2015;8(5):373–384. 10.1007/s40271-015-0118-z 25726010 PMC4575371

[8] Torrance GW Feeny DH Furlong WJ : Multiattribute utility function for a comprehensive health status classification system. Health Utilities Index Mark 2. *Med Care.* 1996;34(7):702–722. 10.1097/00005650-199607000-00004 8676608

[9] Devlin NJ Shah KK Mulhern BJ : A new method for valuing health: directly eliciting personal utility functions. *Eur J Health Econ.* 2019;20(2):257–270. 10.1007/s10198-018-0993-z 30030647 PMC6438932

[10] MVH Group: The measurement and valuation of health: Final report on the modelling of valuation tariffs.Centre for Health Economics, University of York,1995. Reference Source

[11] Richardson JRJ Mckie JR Bariola EJ : Multiattribute Utility Instruments and Their Use.In *Encylopedia of Health Economics.* Elsevier,2014;2:341–357. 10.1016/B978-0-12-375678-7.00505-8

[12] Torrance GW Boyle MH Horwood SP : Application of multi-attribute utility theory to measure social preferences for health states. *Oper Res.* 1982;30(6):1043–1069. 10.1287/opre.30.6.1043 10259643

[13] Torrance GW Furlong W Feeny D : Multi-attribute preference functions. Health Utilities Index. *Pharmacoeconomics.* 1995;7(6):503–520. 10.2165/00019053-199507060-00005 10155336

[14] Rowen D Brazier J Ara R : The role of condition-specific preference-based measures in health technology assessment. *Pharmacoeconomics.* 2017;35(Suppl 1):33–41. 10.1007/s40273-017-0546-9 29052164

[15] Herdman M Gudex C Lloyd A : Development and preliminary testing of the new five-level version of EQ-5D (EQ-5D-5L). *Qual Life Res.* 2011;20(10):1727–1736. 10.1007/s11136-011-9903-x 21479777 PMC3220807

[16] Shah KK Ramos-Goñi JM Kreimeier S : An exploration of methods for obtaining 0 = dead anchors for latent scale EQ-5D-Y values. *Eur J Health Econ.* 2020;21(7):1091–1103. 10.1007/s10198-020-01205-9 32506281 PMC7423806

[17] Dolan P : Modeling valuations for EuroQol health states. *Med Care.* 1997;35(11):1095–1108. 10.1097/00005650-199711000-00002 9366889

[18] Devlin NJ Shah KK Feng Y : Valuing health-related quality of life: An EQ-5D-5L value set for England. *Health Econ.* 2018;27(1):7–22. 10.1002/hec.3564 28833869 PMC6680214

[19] Costa CABE Vansnick JC : The MACBETH Approach: Basic Ideas, Software, and an Application.In: *Advances in decision analysis*. Springer,1999;4:131–157. 10.1007/978-94-017-0647-6_9

[20] Danner M Hummel JM Volz F : Integrating patients' views into health technology assessment: Analytic hierarchy process (AHP) as a method to elicit patient preferences. *Int J Technol Assess Health Care.* 2011;27(4):369–375. 10.1017/S0266462311000523 22004779

[21] Oliveira MD Agostinho A Ferreira L : Valuing health states: is the MACBETH approach useful for valuing EQ-5D-3L health states? *Health Qual Life Outcomes.* 2018;16(1):235. 10.1186/s12955-018-1056-y 30563525 PMC6299594

[22] Oliveira MD Mataloto I Kanavos P : Multi-criteria decision analysis for health technology assessment: addressing methodological challenges to improve the state of the art. *Eur J Health Econ.* 2019;20(6):891–918. 10.1007/s10198-019-01052-3 31006056 PMC6652169

[23] Thokala P Devlin N Marsh K : Multiple criteria decision analysis for health care decision making--an introduction: report 1 of the ISPOR MCDA Emerging Good Practices Task Force. *Value Health.* 2016;19(1):1–13. 10.1016/j.jval.2015.12.003 26797229

[24] Angelis A Kanavos P : Multiple criteria decision analysis (MCDA) for evaluating new medicines in health technology assessment and beyond: the advance value framework. *Soc Sci Med.* 2017;188:137–156. 10.1016/j.socscimed.2017.06.024 28772164

[25] Feeny D Furlong W Torrance GW : Multiattribute and single-attribute utility functions for the health utilities index mark 3 system. *Med Care.* 2002;40(2):113–128. 10.1097/00005650-200202000-00006 11802084

[26] Determann D Lambooij MS Steyerberg EW : Impact of survey administration mode on the results of a health-related discrete choice experiment: online and paper comparison. *Value Health.* 2017;20(7):953–960. 10.1016/j.jval.2017.02.007 28712625

[27] Soekhai V de Bekker-Grob EW Ellis AR : Discrete choice experiments in health economics: past, present and future. *Pharmacoeconomics.* 2019;37(2):201–226. 10.1007/s40273-018-0734-2 30392040 PMC6386055

[28] RStudio Inc: Easy web applications in R.2013. Reference Source

[29] Jonker MF Donkers B de Bekker-Grob E : Attribute level overlap (and color coding) can reduce task complexity, improve choice consistency, and decrease the dropout rate in discrete choice experiments. *Health Econ.* 2019;28(3):350–363. 10.1002/hec.3846 30565338 PMC6590347

[30] Golicki D Jakubczyk M Graczyk K : Valuation of EQ-5D-5L health states in Poland: the first EQ-VT-based study in Central and Eastern Europe. *Pharmacoeconomics.* 2019;37(9):1165–1176. 10.1007/s40273-019-00811-7 31161586 PMC6830402

[31] de Charro F Busschbach J Essink-Bot ML : Some considerations concerning negative values for EQ-5D health states.In *EQ-5D concepts and methods: A developmental history.* Springer,2005;171–179. 10.1007/1-4020-3712-0_14

[32] Ioannidis JP Garber AM : Individualized cost-effectiveness analysis. *PLoS Med.* 2011;8(7):e1001058. 10.1371/journal.pmed.1001058 21765810 PMC3134464

[33] Ramos-Goñi, JM Pinto-Prades JL Oppe M : Valuation and modeling of EQ-5D-5L health states using a hybrid approach. *Med Care.* 2017;55(7):e51–e58. 10.1097/MLR.0000000000000283 25521503 PMC7659432

[34] Johnson EJ Shu SB Dellaert BGC : Beyond nudges: Tools of a choice architecture. *Mark Lett.* 2012;23:487–504. 10.1007/s11002-012-9186-1

[35] Nicolet A Groothuis-Oudshoorn CGM Krabbe PFM : Does inclusion of interactions result in higher precision of estimated health state values? *Value Health.* 2018;21(12):1437–1444. 10.1016/j.jval.2018.06.001 30502788

[36] Green C Brazier J Deverill M : Valuing health-related quality of life. A review of health state valuation techniques. *Pharmacoeconomics.* 2000;17(2):151–165. 10.2165/00019053-200017020-00004 10947338

[37] Attema AE Edelaar-Peeters Y Versteegh MM : Time trade-off: one methodology, different methods. *Eur J Health Econ.* 2013;14 Suppl 1(Suppl 1):S53–64. 10.1007/s10198-013-0508-x 23900665 PMC3728453

[38] Lipman SA Brouwer WBF Attema AE : What is it going to be, TTO or SG? A direct test of the validity of health state valuation. *Health Econ.* 2020;29(11):1475–1481. 10.1002/hec.4131 32744408 PMC7689723

[39] Brazier J Roberts J Deverill M : The estimation of a preference-based measure of health from the SF-36. *J Health Econ.* 2002;21(2):271–292. 10.1016/s0167-6296(01)00130-8 11939242

