# Supplementary material for: The Online Elicitation of Personal Utility Functions (OPUF) tool: a new method for valuing health states
Source: Wellcome Open Res. 2022 Jan 14;7:14. [Version 1] doi: 10.12688/wellcomeopenres.17518.1 (PMC9396078; doi:10.12688/wellcomeopenres.17518.1)
Supplement: Peer review history

# Peer review: Reviewer response for version 1

**DOI:** 10.21956/wellcomeopenres.19370.r51613

**Published:** 2022-08-22

**Authors:** Stefan A. Lipman

**Affiliations:** 1 Erasmus School of Health Policy & Management, Erasmus University Rotterdam, Rotterdam, The Netherlands

This paper is a well-written account of the development of the online personal utility function (OPUF) methodology. The paper is well-written, easy to follow and presents a method that will be a valuable addition to the health economics and outcomes researchers' toolbox. It provides an unprecedented ability to investigate heterogeneity in preferences.

 The OPUF tool is easy-to-use, and beautifully designed. The paper outlines the decision-making that went into the design quite nicely, for example in 3.3 step 3. 

 My comments are mostly minor matters that I found unclear when reading the paper, or thoughts that I had whilst reading. Some matters could be easily clarified in the paper if revised versions are uploaded, some more conceptual matters are for the authors to consider in their continued development of the OPUF method.

Section 2.3: 

- Overall, I found section 2.3 to be quite confusing. This is mostly created by the use of 'objective'. The term lacks definition or examples, but the first paragraph of this section reads as if it is a goal someone may have. It then is highly confusing why dimensions **are **objectives? Examples could help, or doing away with objectives all-together (as I don't think they are key for applications of MAUT?).

Section 2.4:

- It is unclear if this text functions as a way of describing what is standard (which it seems it does at times, but I would not call this a standard way of operationalizing utility functions for health), or whether these are preliminaries needed for your method (which seems the case given that you already commit to an anchoring strategy). 

Section 3.2:

- 'First, we experimented with various approaches for emulating the PUF tasks, that were applied in face-to-face interviews conducted online survey. ' 
  There is a language issue here, but also, some more details may be added about this pre-testing phase. What kind of interviews, with whom etc? 

Section 3.3:

- Step 3: Assuming equidistance between levels across dimensions imposes a restricting assumption on respondents' preferences. Furthermore, the survey imposes consistency in level-ordering on preferences (you can't violate the order of the instrument). 
- Step 5: Why was the importance chosen as order for swing weighting? 
- Step 8: Can we aggregate PUFs when different methods were used to anchor (either the position of dead task or the dead VAS?). Why not use one VAS across all task?

Section 4:

- What were respondents paid? 
- In developing the tool you wrote for step 3 that default values were removed because they caused strong anchoring effects, yet in the quantitative pilot results you write: 'Forty (80%) and 41 (82%) participants set their own values for the slight and severe levels, i.e. they changed the default values. For the moderate level, only 26 (52%) changed the value, which may be an indication for the presence of an anchoring effect.'. When were the default values removed exactly? The way the paper is currently written suggests it was in between the qualitative and quantitative pilot, but the results suggest differently. Given the strong anchoring it implies it seems relevant to make clear.
- How do the swing weights for dimensions correspond to other work in the UK? I think anxiety typically has the largest utility decrements in value sets in Western countries, but here it is least important?
- The accuracy of predicting DCE choices looks impressive but is also difficult to gauge. How good is 80% on three choices? This is hard to say based on the data collected here, but it would be of interest to me to know how good a simple decision rule (e.g. respondents picking the state with the lowest level-sum-score) performs vs. the personal utility functions. Also, if people were filling out the OPUF questions nearly randomly as well as the DCE, we would find high accuracy, but still not high validity. Could this have happened (see also comment on data quality).

Section 5:

 A few points that I feel are worth thinking about and/or discussing:

- Compositional tasks typically involve an error structure (e.g. the error component in random utility models). The authors propose that an utility function can be identified at the individual level, but it is unclear what the role of measurement error is in this framework. At least in TTO-based value set one can hope that error balances out when sufficiently large samples are recruited, but this is not the case when interpreting PUFs at the individual level. I feel this is worth discussing, especially since much of the method is 'chained', i.e. error in the early phases will propagate into subsequent phases. Imagine for example someone who overestimates the 'true' difference between slight and moderate, if such a thing exists, by 10%. This would have huge influence on their PUF. Is there any way to control for this or check for its' occurrence, and if not what is the value of using this in small samples?
- In line with this comment, I feel that the authors should consider or discuss data quality. It appears OPUF should be used as a stand-alone task without interviewers. I commend the authors on developing such an intuitive and easy to navigate survey. All this effort should minimize respondents with the best of intentions misunderstanding the task - yet when using such methods online there is also respondents with different goals: maximizing earning with as little effort as possible. OPUF imposes quite some preference consistency within its' tasks, so what avenues are available to identify respondents that simply were unwilling to engage with the task? OPUF will generate a personal utility function for them, with as far as I can tell no way of determining its' validity. 

Is the rationale for developing the new method (or application) clearly explained?

Yes

Is the description of the method technically sound?

Yes

Are the conclusions about the method and its performance adequately supported by the findings presented in the article?

Yes

If any results are presented, are all the source data underlying the results available to ensure full reproducibility?

Yes

Are sufficient details provided to allow replication of the method development and its use by others?

Partly

Reviewer Expertise:

health state valuation, experiments, behavioural economics.

I confirm that I have read this submission and believe that I have an appropriate level of expertise to confirm that it is of an acceptable scientific standard.

# Peer review: Reviewer response for version 1

**DOI:** 10.21956/wellcomeopenres.19370.r51612

**Published:** 2022-08-04

**Authors:** Ciaran O'Neill

**Affiliations:** 1 Centre for Public Health, Royal Victoria Hospital, Queen's University Belfast, Belfast, UK

The paper discusses a method for constructing health preference weights based on a compositional approach, and presents the results of a pilot online deployment of the approach and discusses the implications of their findings.

 The paper is clearly set out and the methods generally easy to follow.

 The authors identify efficiency as one of the major attributes of their approach - reduced sample sizes being able to provide reasonably precise estimates of preference weights.

 The authors are correct, I think that funders may be somewhat wary of this approach until further work on it has been conducted and the investments in developing protocols similar to those that exist for decompositional preference methods have been developed.

 Regardless, the approach set out provides a road map to examine valuable avenues of research in preference elicitation and measurement. It offers opportunities, for example, to examine the mutability of preference weights as individuals age or encounter "life-changing" events. It offers the opportunity to explore groups changes in preferences that might intersperse larger decompositional exercises where for example a society or sub-group within it has been exposed to an event such as an economic shock, a global pandemic or even a mass shooting. Further work may be necessary before such uses are explored. For example, an examination of test-retest reliability of preference weights might be useful and; examination of preferences among groups who may be less computer literate/highly educated, than those examined here. The paper sets out clearly though, an approach that offers much potential. 

 Minor points:

- I think there is a typographical error in the value function (section 2.4) where the denominator should I think be 10,000.
- In the normalization of weights (section "How to construct PUF from participants' responses") a worked example on weights might be useful to those seeking to replicate results.
- At the risk of sounding pedantic, I disagree with the opening sentence that QALYs are "an essential component in health economic evaluations". They are certainly useful, popular and may be the main way in which outcomes are measured for resource allocation but the use of other outcome measures in cost-effectiveness studies, may remain valid depending on the circumstances of the evaluation.

Is the rationale for developing the new method (or application) clearly explained?

Yes

Is the description of the method technically sound?

Yes

Are the conclusions about the method and its performance adequately supported by the findings presented in the article?

Yes

If any results are presented, are all the source data underlying the results available to ensure full reproducibility?

Yes

Are sufficient details provided to allow replication of the method development and its use by others?

Partly

Reviewer Expertise:

Health economics

I confirm that I have read this submission and believe that I have an appropriate level of expertise to confirm that it is of an acceptable scientific standard, however I have significant reservations, as outlined above.

# Peer review: Reviewer response for version 1

**DOI:** 10.21956/wellcomeopenres.19370.r48438

**Published:** 2022-02-23

**Authors:** Katie Spencer

**Affiliations:** 1 Academic Unit of Health Economics, Leeds Institute of Health Sciences, University of Leeds, Leeds, UK; 2 Leeds Teaching Hospitals NHS Trust, Leeds, UK

The authors present an innovative approach to health state valuation able to identify a personal utility function using a relatively simple online tool (OPUF). The steps of development are laid out in detail and they should be congratulated on the careful and methodical development of the OPUF. Many of my thoughts are really comments and questions of interest, potentially for further work, rather than limitations of the manuscript.

Minor points to modify:

 In section 2.4 I would have found it useful to have a little more clarity in the definition of criteria/dimension weights. Simply referring to the criteria as dimensions in the text here would probably be sufficient. I realise the terminology (“criterion”) used stems from the origin in MAVT/MAUT but coming into this with the EQ-5D in mind I find the word dimension easier to understand. Probably just me being slow on the uptake but possibly more intuitive to those reading this from the EQ-5D perspective.

 Section 3.2: Could you re-phrase the third sentence please?

 Figure 3: It might be worth re-phrasing the figure legend. It sounds like there are more than one thick lines at the moment but I can only see one. Am I missing something?

Comments to consider:

 Was any work done on the individual reproducibility of this? If an individual repeats the OPUF can the model from the first completion accurately predict the responses the second time? It strikes me that this is more important in the compositional approach than it is with the de-compositional one. In a large enough sample for the latter the central estimate might be expected to be reasonably consistent. Here, if the focus is on the individual valuation, whilst the group average may be consistent the individual response may differ. It would be useful to understand the extent to which this occurs and particularly relevant if it were to be used in shared-decision making.

 It is striking that mobility has the highest swing weighting on average and yet “I am unable to walk about” is a normal state for people with a permanent loss of mobility and is readily adapted to. I acknowledge that this is potentially an issue arising from the descriptive system not just the valuation task. However, as with many of these tasks if the respondent focusses on the acute change this state might appear unbearable but with adaptation many will thrive. It would be really interesting to understand how people with different health and disability experiences respond to these tasks both from a qualitative and quantitative perspective. The OPUF would allow this investigation of heterogeneity in a way decompositional methods can’t readily address.

 You mention the difference in the value of ‘55555’ in comparison to Devlin et al. Could you make any comparisons with the mean disutilities in the context of DCE based value sets? It would be interesting to see the comparison. Could they be presented alongside table 3? I accept that with a relatively small sample, differences might reflect the participants involved rather than the differences resulting from the methods.

 You comment on the relatively high educational attainment of the participants. To what extent do you think this will influence the wider acceptability of the OPUF? Could you comment on this please?

 The potential role of this in shared-decision making is fascinating. Definitely needs investigating though. Ww would need to understand if the OPUF is acceptable to patients? Including whether the inclusion of states equivalent to dead are acceptable?

 Having the same level ratings across all dimensions seems likely to be problematic as this isn’t a neutral assumption and might have a significant impact upon the value of levels for some dimensions (certainly in other value sets the levels are not equal across dimensions). Equally, the extent to which it is realistic to think that participants would vary their responses for each dimension separately is impossible to know. It would be interesting (and presumably relatively simple) to test this.

 I accept your point about interaction effects but worry that the relevant interactions here are not so much a challenge for statistical modelling but for the cognitive process of the DCE. This is relevant to other valuation studies as much as the OPUF. There are health states presented which seem unlikely; for example if I am unable to do my usual activities I would expect not to be able to wash or dress myself and probably not to be able to walk around. As a result some of the compared health states are cognitively very difficult to envisage as the imagined health state can’t exist in reality and disentangling the dimensions from the wider state is cognitively challenging. Given the flexibility of the OPUF would it be possible to investigate the consistency of responses for these difficult to envisage states as compared to more easily imagined ones?

Is the rationale for developing the new method (or application) clearly explained?

Yes

Is the description of the method technically sound?

Yes

Are the conclusions about the method and its performance adequately supported by the findings presented in the article?

Yes

If any results are presented, are all the source data underlying the results available to ensure full reproducibility?

Yes

Are sufficient details provided to allow replication of the method development and its use by others?

Yes

Reviewer Expertise:

Health economics in Clinical Oncology; improving the value of routinely delivered care.

I confirm that I have read this submission and believe that I have an appropriate level of expertise to confirm that it is of an acceptable scientific standard.
